# *Colchicum autumnale* in Patients with Goitre with Euthyroidism or Mild Hyperthyroidism: Indications for a Therapeutic Regulative Effect—Results of an Observational Study

**DOI:** 10.1155/2016/2541912

**Published:** 2016-02-03

**Authors:** Christian Scheffer, Marion Debus, Christian Heckmann, Dirk Cysarz, Matthias Girke

**Affiliations:** ^1^Integrated Curriculum for Anthroposophic Medicine, Institute for Integrative Medicine, Faculty for Health, Department for Medicine, Alfred-Herrhausen-Straße 50, 58448 Witten, Germany; ^2^Gemeinschaftskrankenhaus Herdecke, Department for Internal Medicine, Gerhard-Kienle-Weg 4, 58313 Herdecke, Germany; ^3^Research Institute Havelhöhe, Kladower Damm 221, 14089 Berlin, Germany; ^4^Department of Internal Medicine, Havelhöhe Hospital, Kladower Damm 221, 14089 Berlin, Germany; ^5^Institute for Integrative Medicine, Faculty for Health, Department for Medicine, University of Witten/Herdecke, Alfred-Herrhausen-Straße 50, 58448 Witten, Germany

## Abstract

*Introduction*. Goitre with euthyroid function or with subclinical or mild hyperthyroidism due to thyroid autonomy is common. In anthroposophic medicine various thyroid disorders are treated with* Colchicum autumnale* (CAU). We examined the effects of CAU in patients with goitre of both functional states.* Patients and methods*. In an observational study, 24 patients with goitre having suppressed thyroid stimulating hormone (TSH) levels with normal or slightly elevated free thyroxine (fT4) and free triiodothyronine (fT3) (group 1, *n* = 12) or normal TSH, fT3, and fT4 (group 2, *n* = 12) were included. After 3 months and after 6 to 12 months of CAU treatment, we investigated clinical pathology using the Hyperthyroid Symptom Scale (HSS), hormone status (TSH, fT4, and fT3), and thyroidal volume (tV).* Results*. After treatment with CAU, in group 1 the median HSS decreased from 4.5 (2.3–11.8) to 2 (1.3–3) (*p* < 0.01) and fT3 decreased from 3.85 (3.5–4.78) to 3.45 (3.3–3.78) pg/mL (*p* < 0.05). In group 2 tV (13.9% (18.5%–6.1%)) and TSH (*p* < 0.01) were reduced. Linear regression for TSH and fT3 in both groups indicated a regulative therapeutic effect of CAU.* Conclusions*. CAU positively changed the clinical pathology of subclinical hyperthyroidism and thyroidal volume in patients with euthyroid goitre by normalization of the regulation of thyroidal hormones.

## 1. Introduction

Goitre and thyroid nodules are very common diseases, especially in iodine-deficient areas. In Germany, for example, where iodine deficiency was widely spread and has been improved quite recently, a prevalence of more than 30% is still found in some areas [1]. The natural history of goitre is characterised by thyroid growth, nodule formation, and functional autonomy [2]. Goitre as a diffuse thyroid enlargement is more common in younger patients and usually appears with normal or sometimes high TSH where iodine and/or thyroxine are considered for therapy [3]. Thyroid nodules are more common in the elderly, sometimes with functional autonomy resulting in subclinical or overt hyperthyroidism. In the latter case, iodine should be avoided and a symptomatic or thyreostatic therapy is considered as well as definite treatments like operation or radiation [3, 4].

In anthroposophic medicine, a form of integrative medicine [5, 6],* Colchicum autumnale* (CAU) is used for the treatment of various thyroid disorders including goitre. This prescription was first used in the 1920s by Steiner and Wegman in order to stimulate autoregulation of the thyroid [7]. A retrospective study showed positive effects on clinical symptomatology and hormone metabolism in patients with subclinical hyperthyroidism [8]. Hence, in contrast to conventional treatment, where opposite strategies are applied in nontoxic goitre compared to nodular disease with functional autonomy, CAU is used in different functional situations, from (subclinical) hypo- to hyperthyroidism. Here, we performed an observational study (OS) to assess the clinical effects of CAU in patients with thyroid diseases. The OS had three primary goals:

1. Regarding patients with goitre and subclinical or mild hyperthyroidism: is there a positive effect of CAU on clinical symptomatology and on hormone metabolism? (group 1).
2. Regarding patients with goitre: is there a positive effect of CAU on thyroid volume? (groups 1 and 2).
3. Regarding patients with goitre and euthyroidism or (subclinical) hyperthyroidism: is there an adaptive normalization of hormone metabolism indicating a therapeutic regulative effect of CAU? (groups 1 and 2).

## 2. Patients and Methods

### 2.1. Patients

We included all patients in our OS who, over the course of 20 months (in 2001/2002), were treated with CAU for goitre with subclinical or mild hyperthyroidism (group 1) or with euthyroidism (group 2) at the Gemeinschaftskrankenhaus Havelhöhe, Berlin, Germany (see inclusion criteria in Table 1). Patients received CAU as part of their routine care as in- and outpatients of our department for general internal medicine. We restricted our analysis to patients with nontoxic goitre and excluded patients with Graves' disease, Hashimoto's thyroiditis, De Quervain's thyroiditis, and low T3 syndrome by clinical symptomatology, thyroid antibodies (thyroid peroxidase antibodies, thyroid receptor antibodies), and ultrasound scan. To minimise other effects on thyroid function, patients with feverish infections, patients having undergone radio- or chemotherapy as well as those who were exposed to iodine or who had been treated with thyreostatics or TSH-suppressive medications within the last three months prior to the study were also excluded from analysis. Pregnant or breastfeeding patients do not receive CAU in these low potencies to exclude any effect on the child.

We performed an observational study without significant deviation from clinical practice where an ethics approval is usually not required [9]. Patients were informed about the aim of the study, the medication and possible side effects, and their right to refuse participation without negative consequences for their treatment. All patients gave their written informed consent to participate.

### 2.2. Follow-Up and Progress Parameters

**Figure 1:**
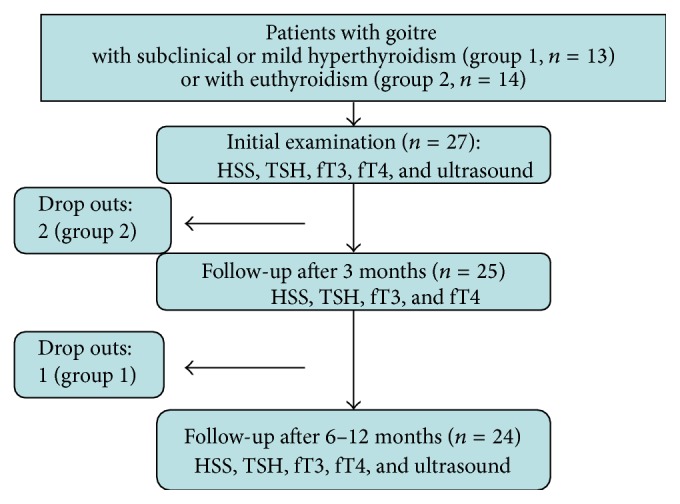
Follow-up. HSS: Hyperthyroid Symptom Scale; fT3: free triiodothyronine, fT4: free thyroxine; TSH: thyroid stimulating hormone.

The observation period was 6 to 12 months for both groups (see Figure 1); progress control tests were carried out after three months.

The Hyperthyroid Symptom Scale (HSS) developed by Klein et al. [10] was used to assess the clinical pathology of hyperthyroidism. This questionnaire contains 10 items, which determine typical symptoms of hyperthyroidism such as nervousness, heat intolerance, sweating, or diarrhoea. Each item scores 0 to 4 points depending on the severity of symptoms. According to Klein et al., patients with overt hyperthyroidism score 20 points or more [10].

TSH was measured via a 3rd generation radioimmune assay with high sensitivity (0.015 mU/L). Thyroid hormones and thyroidal antibodies were also determined via radioimmune assays in an external laboratory.

The thyroid ultrasound was carried out with a 7.5 or 5.0 MHz probe (Siemens, Sonoline Elegra); the determination of volume was based on a formula by Brunn et al. [11].

### 2.3. Treatment

Treatment was carried out with CAU preparations manufactured from the bulb of the plant with a specific rhythmic procedure (“RH preparation”) and potentiated three (D3) or six times (D6) with a 10% dilution. The treatment approach was in accordance with long-term clinical use in AM [8]. Our usual therapeutic proceeding includes changes in potency of CAU according to the following principles: if adverse drug events (ADE) occurred, potency would be changed to RH D6. If no positive effects were seen concerning clinical pathology or thyroid function parameters, preparation would be changed to lower potencies, for example, D2 and D1. According to the manufacturer, CAU RH D3, 3 × 20 drops contained 4.5–9 *μ*g/mL colchicine. Since the RH preparation is only produced in D3 and D6, eD-preparations (ethanolic preparation, a special heating process at 37°C) had to be used for lower potencies (D2, D1).

### 2.4. Statistical Analysis

All statistical computations were performed with the Statistical Package for the Social Sciences (SPSS) for Macintosh (Version 22.0). Due to limited number of participants, nonparametric statistical procedures were used. The Wilcoxon Test was used to compare the progress parameters before and after treatment. Spearman's rank correlation was calculated to determine the relationship between laboratory chemical and clinical parameters. Statistical significance was always calculated bilaterally. A *p* value < 0.05 was considered statistically significant. Missing values were replaced with the last measured values.

Application of CAU is intended to stimulate the autoregulation of involved physiologic processes (e.g., the thyroid function). Thus, one should expect normalization of involved functions. A normalization is characterised by two features:

1. The progress parameters tend toward a value within the normal range.
2. The direction and strength of these changes depend on the initial value.

This means the high values of fT3 should decrease more than slightly elevated ones while low values should increase. Linear regression was used to quantify normalization [12, 13]. The difference of a parameter between two measurements was plotted against initial values for each subject. The determination coefficient (*r* ^2^) was calculated to check whether there was a significant relationship between the difference of a parameter against the initial value. Determination coefficient *r* ^2^ = 0 indicates no and *r* ^2^ = 1 a perfect correlation, that is, a normalization of involved physiologic functions. The probability of rejecting the null hypothesis of no relationship was analysed and *p* < 0.05 was considered as statistically significant.

## 3. Results

### 3.1. Patients

27 patients were included (see Figure 1); three of them did not reach the minimum follow-up of six months. One patient stopped treatment after one month, another patient after two months, and a third after three months, all on their own accord. They stated lack of motivation due to insufficient or nonexistent clinical pathology. The characterisation of patients with regards to gender, age, diagnosis, concomitant diseases, and concomitant medication is shown in Table 2. As expected, there were more females (*n* = 21) than males (*n* = 3); the median age was 49.5 years. Amongst the 15 patients with concomitant diseases, cardiovascular disease (*n* = 9) was the most common, particularly arterial hypertension (*n* = 7) and arrhythmia (*n* = 5). More than half of the patients (*n* = 15) were treated with conventional medication; a third took further homeopathic or specific anthroposophic remedies (*n* = 8). Beta blockers were the most common drugs (*n* = 6) followed by other antihypertensives (*n* = 6). Compared to group 1, euthyroid patients were younger (median age 44 versus 63 years) and their thyroidal volume showed less interquartile range (35.3 to 57.8 versus 38 to 73 mL).

### 3.2. Adverse Drug Events and Patient Reported Effects

No severe adverse drug events (ADE) were documented during the observed treatment period.

A total of four patients reported negative effects (see Table 3) such as fatigue, palpitations, sweating, and increased agitation. In three cases these symptoms decreased when the preparation was changed from RH D3 to RH D6 and for one patient the medication was stopped (after 8 months).

14 patients reported positive effects on their health during treatment. The most common feedback was increased inner calm and equilibrium (*n* = 12), less of a feeling of having a lump in one's throat (*n* = 7), antidepressive effects, decreased tachycardia, and improved ability to concentrate (each 2x).

### 3.3. Hyperthyroid Symptom Scale

When the HSS was evaluated, there was a significant reduction at both follow-ups, but the subgroup analysis showed different results (see Table 4):

(i) For group 1, one patient had a score of 20 at the initial examination; another three patients had values ≥10. After 3 and 6–12 months, respectively, only one patient had an HSS score of 10 or more. All in all, the median decreased from 4.5 (quartiles 2.3–11.8) at initial examination to 2 (1.3–5) after three months and to 2 (1.3–3) after 6–12 months (Table 4). The decrease was significant at both points in time (*p* < 0.05 at t3, *p* < 0.01 at t6–12).
(ii) For group 2, patients showed a median HSS of 5.5 (2–12.5) at the beginning, with no significant reduction at both follow-up times. There was no patient with a score of 20 or more, but five with ten or more. At the final follow-up three patients scored ten or more points.

### 3.4. Hormone Parameters

During treatment the mean TSH level developed differently in both groups (see Table 4): in group 1 we observed no significant changes; in group 2 there was a significant reduction from 1.2 (0.75–1.73) to 0.85 (0.53–1.38) mU/L after 6–12 months.

The median fT3 level decreased in group 1 from 3.85 (3.5–4.78) pg/mL at initial examination to 3.45 (3.3–3.78) pg/mL at the final follow-up (see Table 4). All three patients, who were suffering from a mild hyperthyroidism (fT3 = 4.8–5.4 pg/mL) at the beginning, achieved normal levels (fT3 = 3.8–4.3 pg/mL) during treatment within three months.

For fT4, there was neither a significant change for the group as a whole nor for subgroups 1 or 2 (see Table 3).

**Figure 2:**
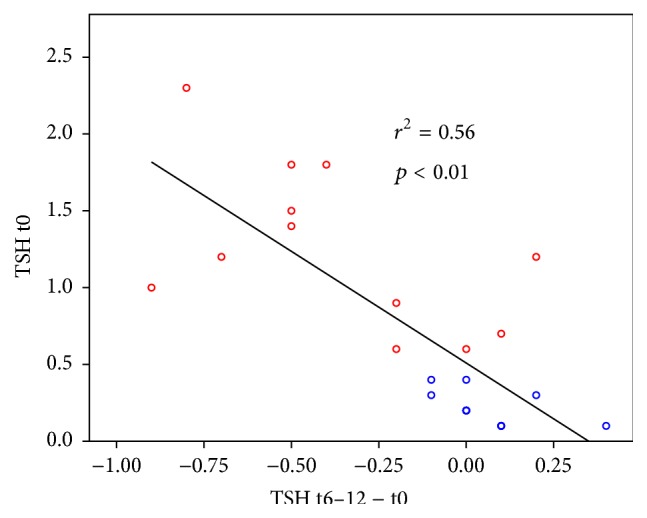
Regression curve of thyroidea stimulating hormone change after 6–12 months (TSH t6–12 − t0) against initial values (TSH t0); *r* ^2^ = determination coefficient: proportion of variance that is declared by the regression curve. Patients with higher initial TSH (TSH t0 > 0.4 mU/mL, group 1: red marks) tend to have significant reductions of TSH (TSH t6–12 − t0) while those with TSH of 0.4 or less (group 2: blue marks) show only small and predominantly positive changes (three blue marks represent two patients).

**Figure 3:**
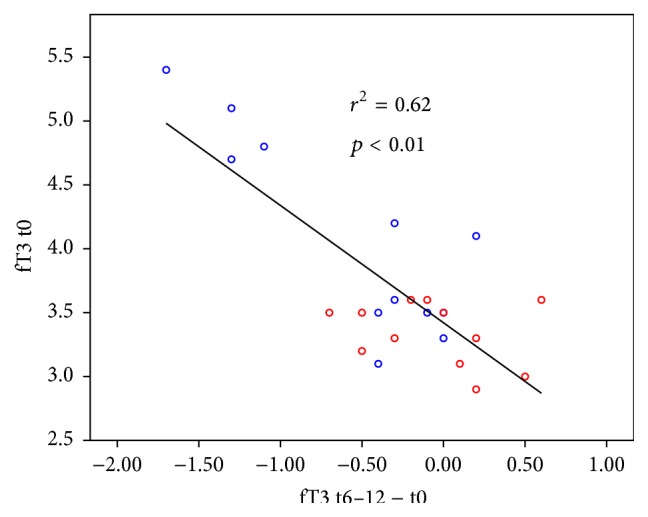
Regression curve of free triiodothyronine change after 6–12 months (fT3 t6–12 − t0) against initial values (fT3  t0); *r* ^2^ = determination coefficient: proportion of variance that is declared by the regression curve. Patients with higher initial fT3 (fT3  t0 > 4.5 mU/mL) tend to have significant reductions of fT3 (fT3 t6–12 − t0 > 1,00 mU/mL) while those with fT3 of 3.5 or less show only small changes (blue marks: group 1; red marks: group 2).

Linear regression showed positive correlations (*p* < 0.01) for TSH and fT3 when final values where analysed against initial ones (see Figures 2 and 3).

### 3.5. Thyroid Volume

Thyroid volume showed no significant change in group 1, but in group 2 a median volume reduction of 13.9% was measured with 8 of 12 patients showing a greater than 10% reduction. A subjective reduction of feeling a lump in one's throat was reported in 50% of patients in group 2 compared to once in group 1.

## 4. Discussion

This OS is the first prospective observational study to examine the effects of CAU on patients with nonimmunogenic goitre. The main focus of the study was the question whether treatment with CAU would stimulate a therapeutic regulative process with adaptive normalization. Therefore, we followed two subgroups with different thyroidal functions to see whether the effect of CAU would be in dependence on the initial value and towards normal range.

In patients with subclinical or mild hyperthyroidism we found a positive effect on clinical symptomatology: most of the patients (75%) described more inner calm and stability and the HSS was reduced at both follow-ups (*p* < 0.05 and 0.01). TSH showed no significant changes whereas fT3 was reduced at both follow-ups.

While functional scintigraphic imaging was not part of the routine care of our patients, it can be assumed that after exclusion of immunogenic and exogenous causes (see exclusion criteria), functional autonomy is the main reason for subclinical or mild hyperthyroidism [14]. Compared to patients with Grave's disease, where an undulating disease is common, patients with nodular goitre or autonomous nodules usually have a persistent or progressive disease with a rate for progression to overt hyperthyroidism of 5–8% per year [15]. Hence, in our cohort, where TSH suppression was caused by nodular goitre or autonomous nodules, one would expect a progress towards hyperthyroidism and neither a reduction of clinical symptoms nor of fT3. Our results are in concordance with the first retrospective study on the effect of CAU [8] where a reduction of thyrotoxic symptoms was seen as well as a decrease of fT3. It may also be of interest that colchicine seems to have a positive effect in patients with Graves' ophthalmopathy [16].

In patients with euthyroid goitre, we found a significant reduction of thyroid volume which was accompanied by a reduced feeling of having a lump in one's throat reported by half of the patients. The observed significant reduction of TSH may be of pathophysiologic relevance since it has been shown that the treatment effect of thyroxin in patients with goitre depends on the degree of TSH suppression [17, 18]. A median volume reduction of around 14% is above the usual interobserver variation of 10% [19]. While therapies with iodine or thyroxin have shown a greater volume reduction of 15–40% [19] in patients with diffuse goitre, studies on patients with nodular goitre showed a smaller volume decrease ranging from 1% [20] to 25% [21] with a lower decrease in patients older than 40 years [22]. Hence, with respect to our patients, where all except one had nodular goitre and a median age of 49.5 years, the measured volume decrease seems to be not much smaller than the usual therapy with thyroxin.

In conclusion, the subjective improvement (only in group 2 but not in 1) and the reduction of the TSH levels support the hypothesis that CAU may have a moderate volume reducing effect in patients with euthyroid goitre. While different in vitro studies have shown an inhibiting effect of colchicine on the morphogenesis of the thyroid follicular [23, 24], our study is the first in vivo study that implicates a possible inhibiting effect on goitre growth.

Regarding the supposed autoregulative effect, we found adaptive normalization concerning fT3 and TSH. fT3 was reduced more in patients with higher initial values but less or not in others, whereas TSH was reduced only in patients with euthyroid goitre but not in those with suppressed TSH. Thus, the normalization of both involved hormones supports the hypothesis of a stimulated autoregulation that directs different processes (e.g., hormone secretion and metabolism) towards the normal range [12, 13]. Adaptive normalization is a known effect of nonspecific interventions like rehabilitation or physical activity for different physiological processes like blood pressure regulation. A similar effect of normalization has been shown for another anthroposophic remedy concerning cardiorespiratory interactions [25].

The ADE reported by patients with regards to restlessness and fatigue could also be interpreted as thyreotoxic symptoms. However, the reversibility observed after the change from the potency of D3 to D6 in the treatment of two patients indicates a possible link to the administration of the drug. On the whole, CAU was well tolerated and did not cause any severe ADE. The used dosage of 3 × 20 drops CAU RH D3 daily seems to be effective, but further studies are necessary to determine the best therapeutic dose and strategy. Since reported symptoms were more present in patients of group 1 with low potencies (D3), it may also be a reasonable strategy to begin with D6 in patients with functional autonomy and symptoms of hyperthyroidism.

Pharmaceutical research usually starts with a few years of preclinical studies with in vitro experiments followed by different phases of clinical trials. This course of action is so costly and time-consuming that it can often only be managed by big pharmaceutical companies. In complementary and alternative medicine, where pharmaceutical companies are usually much smaller but many different therapeutic interventions are already widely used, observational studies are seen as an important instrument to begin with clinical studies and to formulate hypotheses that should be tested in subsequent experiments [26, 27].

## 5. Conclusion

In conclusion, our observational study supports the idea of a stimulation of autoregulatory processes by CAU in patients with goitre with euthyroid function or subclinical hyperthyroidism. Due to methodological limitations of a single arm study, definite statements cannot be made at this stage, but our results support the hypothesis that CAU might be a therapeutic option in patients with goitre. Further studies with more patients and some distinct comparison groups are required.

## Previous Publications

A preliminary analysis of results was presented at the European Conference for Integrative Medicine in 2009. It included results of patients of group 1 complemented with patients with sH due to functional autonomy without goitre that took CAU for three-month duration. The corresponding abstract has been published [28].

## Figures and Tables

**Table 1:** Inclusion criteria.

Group 1: goitre and subclinical or mild hyperthyroidism	Group 2: euthyroid goitre
(i) Thyroid volume > 18 mL (female), >25 mL (male) (ii) TSH ≤ 0.4 mU/L (iii) Peripheral euthyroidism (fT3: 2.2–4.7 pg/mL; fT4: 0.6–1.8 ng/100 mL) or mild hyperthyroidism (fT3 ≤ 6 pg/mL, fT4 < 2.5 ng/100 mL)	(i) Thyroid volume > 18 mL (female), >25 mL (male) (ii) TSH: 0.5–4 mU/L (iii) Peripheral euthyroidism (fT3: 2.2–4.7 pg/ml; fT4: 0.6–1.8 ng/100 ml)

Age > 18	

TSH: thyroid stimulating hormone, fT3: free triiodothyronine, and fT4: free thyroxine.

**Table 2:** Characterisation of patients.

	Group 1	Group 2	Groups 1 and 2
Gender			
Female	10	11	21
Male	2	1	3

Age (years) median (quartiles)	63 (38 to 73)	44 (35.3 to 57.8)	49.5 (38 to 71.8)

Sonographic thyroid volume (mL)	27.7	27	27.1
Median (quartiles)	(19.5 to 57.2)	(23.1 to 42.1)	(21.3 to 43.7)

Diffuse goitre		1	1
Nodular goitre	12	11	23

Euthyroidism		12	12
Subclinical hyperthyroidism	9		9
Hyperthyroidism	3		3

No concomitant diseases	**4**	**5**	**9**
Concomitant diseases	**8**	**7**	**15**
Of circulatory system	5	4	9
Of respiratory system	4	1	5
Of gastrointestinal system	4	1	5
Of muscular-skeletal system	0	1	1
Of endocrine and metabolic system	1	2	3
Of blood and hemopoietic organs	1	0	1
Of gynaecological system	1	0	1
Of nervous system	2	3	5
Psychiatric and behavioural disorders	1	3	4
Of ophthalmic system	0	2	2

No concomitant medication	**4**	**5**	**9**
Concomitant medication	**8**	**7**	**15**
Conventional medical treatment	3	5	8
*β*-blockers	4	2	6
Further antihypertensive agents	3	3	6
Others	5	3	8
Herbal preparations	0	1	1
Homeopathic/anthroposophic medication	5	3	8

**Table 3:** Patient reported effects.

	Group 1	Group 2	Groups 1 and 2
No reported effects	**5 (42%)**	**3 (25%)**	**8 (33%)**

Positive reported effects	**6 (50%)**	**8 (75%)**	**14 (63%)**
More inner calm and equilibrium	4	8	12
Less of feeling a lump in one's throat	1	6	7
Less tachycardia	1	1	2
Less depressive		2	2
Better concentration	1	1	2
Others (one each: decreased hair loss, more weight, more active, and voice more clear)	2	2	4

Negative reported effects^1,2^	**2 (16%)**	**2 (16%)**	**4 (16%)**
Fatigue	2	1	3
Palpitations		1	1
Sweating		1	1
Agitated		1	1

^1^One patient in group 1 and two in group 2 reported negative and positive effects.

^1,2^Reported negative effects diminished after elevation of potency (3 patients) or stopping medication (1x).

**Table 4:** Hyperthyroid Symptom Scale (HSS), thyroid stimulating hormone (TSH), free triiodothyronine (fT3), and free thyroxine (fT4) before (t0), three (t3), and six to twelve (t6–12) months of treatment with *Colchicum autumnale*.

	t0 median (25–75%)	t3 median (25–75%)	t6–12 median (25–75%)	*p* (t3–>t0)	*p* (t6–12–>t0)
HSS					
Group 1	4.5 (2.3 to 11.8)	2 (1.3 to 5)	2 (1.3 to 3)	<0.05	<0.01
Group 2	5.5 (2 to 12.5)	5 (1.3 to 8.8)	5.5 (1 to 13.5)	n.s.	n.s.
Groups 1 and 2	5 (2 to 11.8)	3 (1.3 to 7.5)	2 (1 to 9.8)	<0.05	<0.05

TSH (mU/L)					
Group 1	0.2 (0.1 to 0.3)	0.2 (0.13 to 0.28)	0.2 (0.2 to 0.38)	n.s.	n.s.
Group 2	1.2 (0.75 to 1.73)	0.85 (0.6 to 1.15)	0.85 (0.53 to 1.38)	<0.01	<0.01
Groups 1 and 2	0.5 (0.2 to 1.2)	0.4 (0.2 to 0.88)	0.45 (0.2 to 0.88)	<0.05	(<0.1)

fT3 (pg/mL)					
Group 1	3.85 (3.5 to 4.78)	3.4 (3.2 to 3.85)	3.45 (3.3 to 3.78)	<0.05	<0.01
Group 2	3.4 (3.13 to 3.58)	3.3 (3 to 3.5)	3.3 (3 to 3.5)	n.s.	n.s.
Groups 1 and 2	3.5 (3.3 to 3.98)	3.35 (3.13 to 3.6)	3.4 (3.1 to 3.65)	(<0.1)	<0.05

fT4 (ng/100 mL)					
Group 1	1.15 (0.93 to 1.35)	1.1 (0.93 to 1.35)	1.1 (1 to 1.2)	n.s.	n.s.
Group 2	1.1 (1.03 to 1.35)	1.1 (1 to 1.28)	1.1 (0.9 to 1.28)	n.s.	n.s.
Groups 1 and 2	1.1 (1 to 1.35)	1.1 (1 to 1.3)	1.1 (1 to 1.2)	n.s.	n.s.

Thyroid volume (mL)			Volume change (t6–12 − t0) %		
Group 1	27.7 (19.5 to 57.2)		+7 (−0.2 to +15.5)		n.s.
Group 2	27 (23.1 to 42.1)		−13.9 (−18.5 to −6.1)		<0.01
Groups 1 and 2	27.1 (21.3 to 43.7)		−3.8 (−14.7 to +8.7)		n.s.

## Abbreviations

ADE: Adverse drug events
CAU: *Colchicum autumnale*
eD: ethanolic Digestio
fT3: Free triiodothyronine
fT4: Free thyroxine
HSS: Hyperthyroid Symptom Scale
RH preparations: Preparations treated with rhythmical procedures
MNG: Multinodular goitre
MV: Mean value
OS: Observational study
*r*^2^: Determination coefficient
RTM: Regression to the mean
Sd: Standard deviation
sH: Subclinical hyperthyroidism
TA: Toxic adenoma
TSH: Thyroid stimulating hormone.
